# A Rare Case of Horse Shoe Shaped Lipoma of the Upper Extremity

**Published:** 2013-01

**Authors:** Nikhil Panse, Parag Sahasrabudhe, Ajay Chandanwale, Namrata Joshi

**Affiliations:** 1 Department of Plastic Surgery, BJ Med- ical College and Sassoon Hospital, Pune, India;; 2 Department of Orthopaedics, BJ Medi- cal College and Sassoon Hospital, Pune, India;; 3 Department of Surgery, BJ MedicalCollege and Sassoon Hospital, Pune, India

**Keywords:** Horse shoe shaped lipoma, Upper extremity

## Abstract

Horse shoe shaped lipoma of the upper extremity is a very rare entity. We present a case of 45 years old female who presented with painless progressive swelling over the distal forearm and tingling and numbness over the ulnar nerve territory. MRI and surgical exploration showed a horse shoe shaped multilobulated lipoma encasing the distal ulna. The mass was excised in toto, and the sensory alterations were completely relieved at three months follow up. We would like to highlight this rare occurrence of a horse shoe lipoma and present a detailed history of this case to increase awareness amongst clinicians regarding this condition.

## INTRODUCTION

Lipomas were once believed to be rare in the upper extremity but are now considered common among soft tissue tumors of the hand.^1^ Most often found in subcutaneous fascia, lipomatous neoplasm’s occasionally occur in deeper layers. Development typically begins with an initial insidious growth period followed by a prolonged and latent maintenance state.^2^ Rarely, lipoma may present in an unusual fashion, which might result in lesions entangled within neurovascular structures, and may also result in incomplete excision of the deeper part of the lesion.^3^ In patients with suspected complicated anatomical infiltration or multilocular lesions, or lesions that are deceptively large MR imaging is a useful tool for preoperative evaluation, identification of important structures and planning incisions.^3^^,^^4^ Although rare, abnormal extensions of the lipoma in the upper extremity must be kept in mind while dealing with such lesions. A rare case of multilobulated horse shoe lipoma encasing the distal ulna is highlighted.

## CASE REPORT

**Fig. 1:**
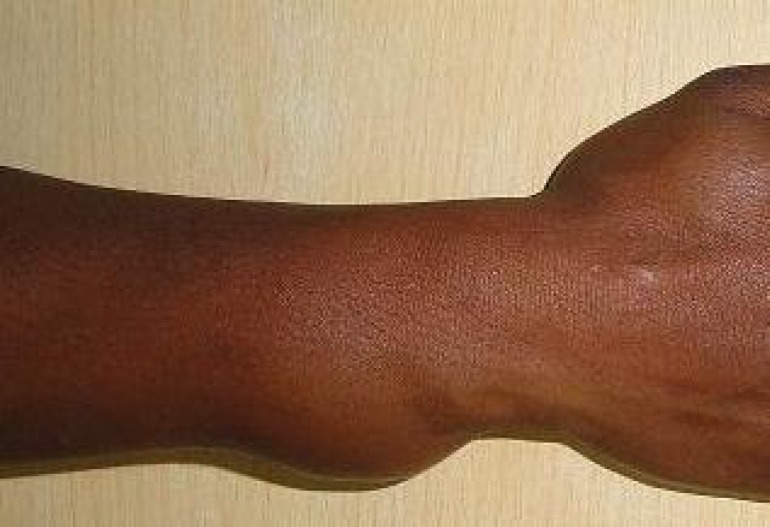
Gross appearance of bulged tumor

A 45 years old lady presented with history of tingling and numbness at palmar aspect of fingertips in the ulnar nerve territory of the right hand since the past four months. She also complained of an asymmetric progressive swelling of her right distal forearm since the past two years. Physical examination revealed a soft tissue swelling localized to the ulnar border of the distal forearm extending volarwards and dorsally. The mass was non-tender and did not transilluminate. The grip strength, adduction and abduction and range of motion were symmetrical in both the hands (Figure 1).

**Fig. 2:**
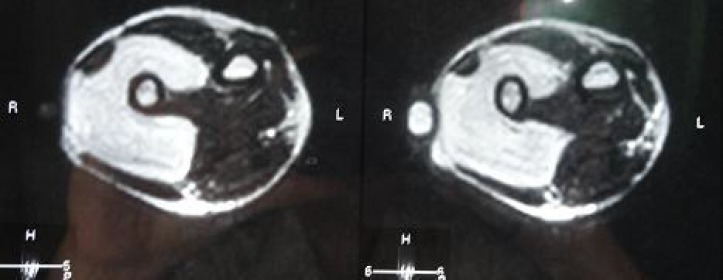
MRI revealed a horse shoe soft tissue mass encasing the distal ulna

MRI showed a well defined horse shoe shaped lobulated mass with homogenous appearance encasing the entire distal ulna (Figure 2). The mass bulged forwards to stretch the flexor carpi ulnaris (FCU) and the extensor carpi ulnaris without actual infiltration of the muscles. The mass measured 10x5.5x4.5 Cm, and showed high signal intensity on T1 and T2 weighted images and low signal intensity on fat suppression images. The ulnar neurovascular bundle appeared largely uninvolved.

**Fig. 3:**
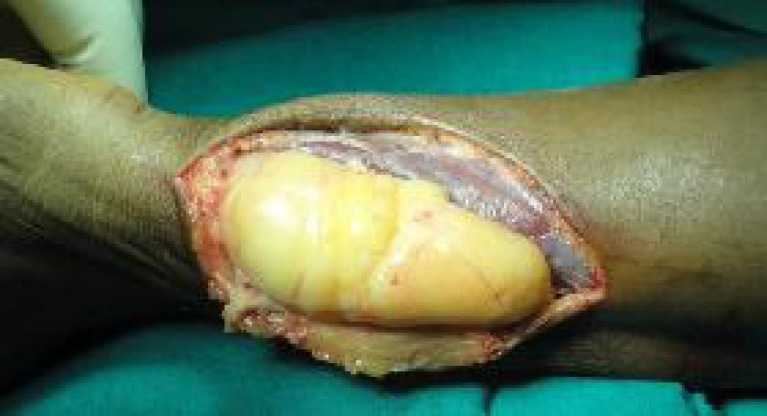
Lipomatous appearance of the tumor

Surgical excision was done under brachial block with tourniquet control. Longitudinal incision was made on the ulnar border of the distal forearm. The FCU was stretched, and displaced outwards. On reflecting the FCU, the mass was exposed (Figure 3).

**Fig. 4:**
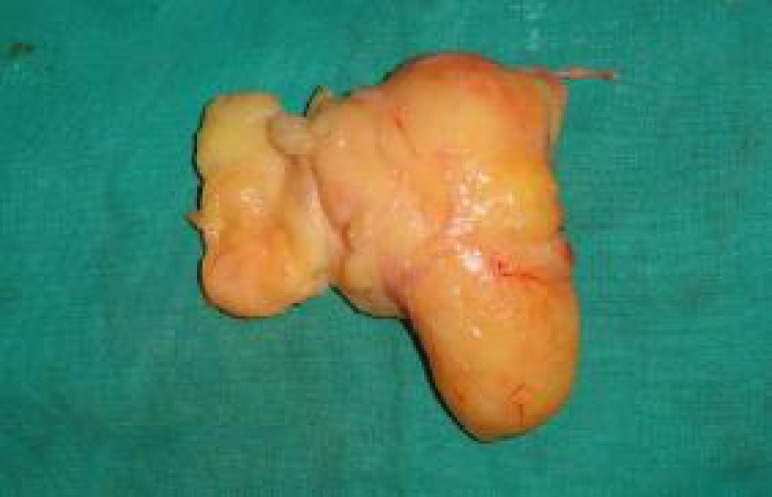
Resected lipoma on the table.

Grossly, the mass was yellowish in color, lipomatous and multilobulated. The mass was gradually dissected (Figure 4). It extended from interosseous membrane on volar side to the interosseous membrane on the dorsal side encasing the entire distal ulna. As the ulnar neurovascular pedicle was at some distance from the mass and appeared largely uninvolved, no further exploration or neurolysis was considered.

Histology was suggestive of lipoma with presence of well differentiated adipocytes. The post operative course was uneventful, and the tingling numbness gradually improved over a period of one month. At three months follow up, there was no evidence of any sensory alterations, and the patient was satisfied.

## DISCUSSION

There is adequate literature on compressive neuropathy caused by giant lipoma in the upper extremity.^1^,^2^ However; to the best of our knowledge and literature search a horse shoe shaped lipoma encasing the entire distal ulna causing compressive neuropathy is not yet reported. We report this case as an extremely rare finding regarding the anatomic extensions of a single giant lipoma in a horse shoe pattern. It should serve to remind the amateur hand surgeon about the abnormal patterns of extension in which a lipoma may present itself in the upper extremity. Whenever a lipoma or recurrent lipoma of the upper extremity is encountered, a horseshoe lipoma must be kept in mind and investigated accordingly It is important to note that MRI is the most significant modality which is helpful in the surgical planning of a symptomatic giant lipoma of the upper extremity.^1^ These tumors may be deceptively large, and preoperative evaluation using MRI will be of help in planning of the incisions and identification of important regional structures that are often displaced or obscured. Using careful surgical techniques, complication rates can be minimized.

Horseshoe lipoma is a rare entity, and must be kept in mind as a differential diagnosis of soft tissue masses and recurrent tumors of the upper extremity. Thorough clinical evaluation supplemented with MRI gives us precise guidelines to the surgical approach to the management of these tumors.

## CONFLICT OF INTEREST

The authors declare no conflict of interest.

## Footenote

Please cite this paper as:

Panse N, Sahasrabudhe P, Chandanwale A, Joshi N. A Rare Case of Horse Shoe Shaped Lipoma of the Upper Extremity. World J Plast Surg 2013;2(1): 41-43.
